# Perioperative laboratory profiles predict complications after extensive head and neck reconstruction: a proof-of-concept study

**DOI:** 10.3389/fonc.2025.1724846

**Published:** 2026-01-02

**Authors:** Tatjana Khromov, Simon Breier, Ulrich Stefenelli, Boris Schminke, Julie Schanz, Andreas Fischer, Henning Schliephake, Phillipp Brockmeyer

**Affiliations:** 1 Department of Oral and Maxillofacial Surgery, University Medical Center Goettingen, Goettingen, Germany; 2 Institute of Clinical Chemistry, University Medical Center Goettingen, Goettingen, Germany; 3 Chi^2^Data Analytics and Artificial Intelligence GmbH, Wuerzburg, Germany; 4 Institute of Clinical Chemistry, Mannheim Medical Center, Heidelberg University, Mannheim, Germany

**Keywords:** head and neck surgery, perioperative laboratory trends, postoperative complications, reconstructive and plastic surgery, risk stratification

## Abstract

**Introduction:**

Surgical reconstruction of head and neck defects after oncological resection is a complex procedure often associated with unpredictable postoperative complications. Hence, laboratory parameter profiles are of considerable interest as potential perioperative predictors.

**Methods:**

This retrospective study analyzed a comprehensive set of laboratory parameters in 233 patients with oral squamous cell carcinoma (OSCC) who underwent tumor resection and reconstruction.

**Results:**

The overall complication rate was 30%, with wound dehiscence (12.4%), pulmonary embolism (PE, 11.6%), and surgical revision (10.3%) being the most common complications. Dynamic analysis of perioperative laboratory parameters from one week before to 49 days after surgery revealed that patients who developed complications showed less pronounced decreases in hemoglobin, hematocrit, and erythrocyte levels. These patients also exhibited altered coagulation and electrolyte profiles. Statistical analysis using logistic regression identified hematocrit slope as independent predictor. Meanwhile, random forest modeling highlighted INR and aPTT as key markers. Subgroup analysis showed that PE, the most clinically significant complication, was associated with abnormal potassium, urea, and protein profiles. Whereas a therapy-related increase in aPTT was observed postoperatively, INR alterations were already evident preoperatively. Conversely, local complications such as wound dehiscence, surgical revision, and graft failure were more strongly associated with deteriorating hematological parameters.

**Discussion:**

Given their multifactorial nature, influenced by comorbidities, tumor biology, and perioperative management, these findings highlight the need for longitudinal laboratory monitoring and prospective validation in controlled settings. Integrating dynamic laboratory trends into multimodal prediction models may facilitate earlier risk stratification and improve individualized perioperative management in head and neck reconstructive surgery.

## 1 Introduction

Due to the region’s anatomical complexity and functional importance, reconstruction of soft and bony tissue defects in the head and neck region following ablative procedures for malignancy, trauma, infection, or congenital anomalies presents a significant surgical challenge (1). Advances in microvascular free tissue transfer have significantly improved reconstructive outcomes by enabling the restoration of form and function (2). A wide variety of free flaps are now routinely used, each of which offers specific advantages depending on the clinical context (3).

While the radial forearm free flap (RFFF) (4), and the anterolateral thigh flap (ALT flap) (5) are widely regarded as the gold standard for soft tissue reconstruction, bony reconstruction of the mandible and maxilla, is commonly performed using the free fibula flap (FFF) (6). Integrating virtual surgical planning (VSP) and computer-aided design/computer-aided manufacturing (CAD/CAM)-guided osteotomies has significantly improved the accuracy and predictability of complex bone reconstructions (7, 8).

Postoperative complications in head and neck reconstruction are typically classified as systemic or local (2, 9–16). Pulmonary complications are among the most frequent systemic events, with reported rates of up to 37%, including serious pulmonary events in up to 33% of cases (11). A pulmonary embolism (PE) is a serious complication of major surgery, especially for immobilized patients. The risk increases with the extent of the surgery and with comorbidities such as obesity, advanced age, and malignancy (17). Important laboratory indicators following postoperative PE encompass D-dimer, C-reactive protein, the neutrophil-to-lymphocyte ratio, and cardiac biomarkers like N-terminal pro-B-type natriuretic peptide and troponin I (18–20). From a prognostic perspective, the ratio of D-dimer to troponin I and the red cell distribution width to platelet ratio offer particularly valuable insights (18, 19). Cardiac events such as myocardial infarction occur in approximately 6.6% of patients (13), while infectious complications - particularly pneumonia (up to 37.5%) and sepsis (up to 26.5%) - are prevalent (13). Other systemic issues include alcohol withdrawal and embolic phenomena, contributing to overall systemic complication rates ranging from 14% to 20.5% (2, 9–16).

Local complications predominantly involve flap-related issues, including partial or total flap loss (0.8–16.2%) and wound infections (1.6–53.7%) (2, 9–11). Donor or recipient site complications may require reoperation, with reintervention rates reported as high as 88.4% in some series (11, 12). Risk factors consistently associated with higher complication rates include advanced age (≥70 years), elevated comorbidity indices (e.g., ASA classification, Charlson Comorbidity Index), alcohol abuse, prolonged surgery time, increased blood loss, and reconstruction for advanced-stage tumors (T4) (2, 9–16). These complications are closely linked to longer intensive care unit (ICU) and hospital stays, as well as increased healthcare costs (2, 9–16).

To enhance risk stratification and guide perioperative management, recent studies have focused on the predictive value of laboratory-based parameters. Serum albumin has emerged as a reliable marker, with hypoalbuminemia significantly associated with adverse outcomes (21). Similarly, postoperative hypoproteinemia increases complication risk, particularly in elderly patients undergoing RFFF or ALT flap reconstruction (22, 23). The Prognostic Nutritional Index (PNI) - a composite of albumin and lymphocyte count - has been validated as a predictor of 30-day postoperative complications in patients ≥60 years undergoing free flap reconstruction for OSCC, either alone or in combination with the Modified Frailty Index (5-mFI) (24). Hemoglobin levels have also been linked to complications, although threshold definitions vary widely across studies (22, 23). The Hemoglobin-Albumin-Lymphocyte-Platelet (HALP) score, by contrast, has demonstrated strong predictive accuracy (AUC 0.85) for complications such as flap necrosis, infection, fistula, and hematoma in head and neck free flap surgery (25). This composite index provides a broader reflection of physiological reserve than individual parameters alone. Notably, higher platelet counts have been correlated with improved donor site healing, underscoring their role in tissue regeneration both independently and as part of the HALP score (26). Inflammatory markers such as C-reactive protein (CRP) and white blood cell count (WBC), by contrast, appear to have limited predictive utility compared to nutritional and composite indices (26). Sarcopenia has also been identified as a relevant predictor of complications and may offer a more objective assessment of nutritional status than albumin, which is often confounded by tumor-associated inflammation (27).

Given the multifactorial nature of postoperative complications, recent attention has turned to machine learning models that integrate laboratory and clinical variables to improve prediction of unplanned readmissions and reoperations in head and neck free flap reconstruction (28, 29). In this context, the incorporation of laboratory-based risk stratification into perioperative protocols holds considerable promise for improving complication prediction, supporting clinical decision-making, and ultimately optimizing outcomes in these complex surgical patients.

## 2 Material and methods

### 2.1 Study design and population

**Figure 1:**
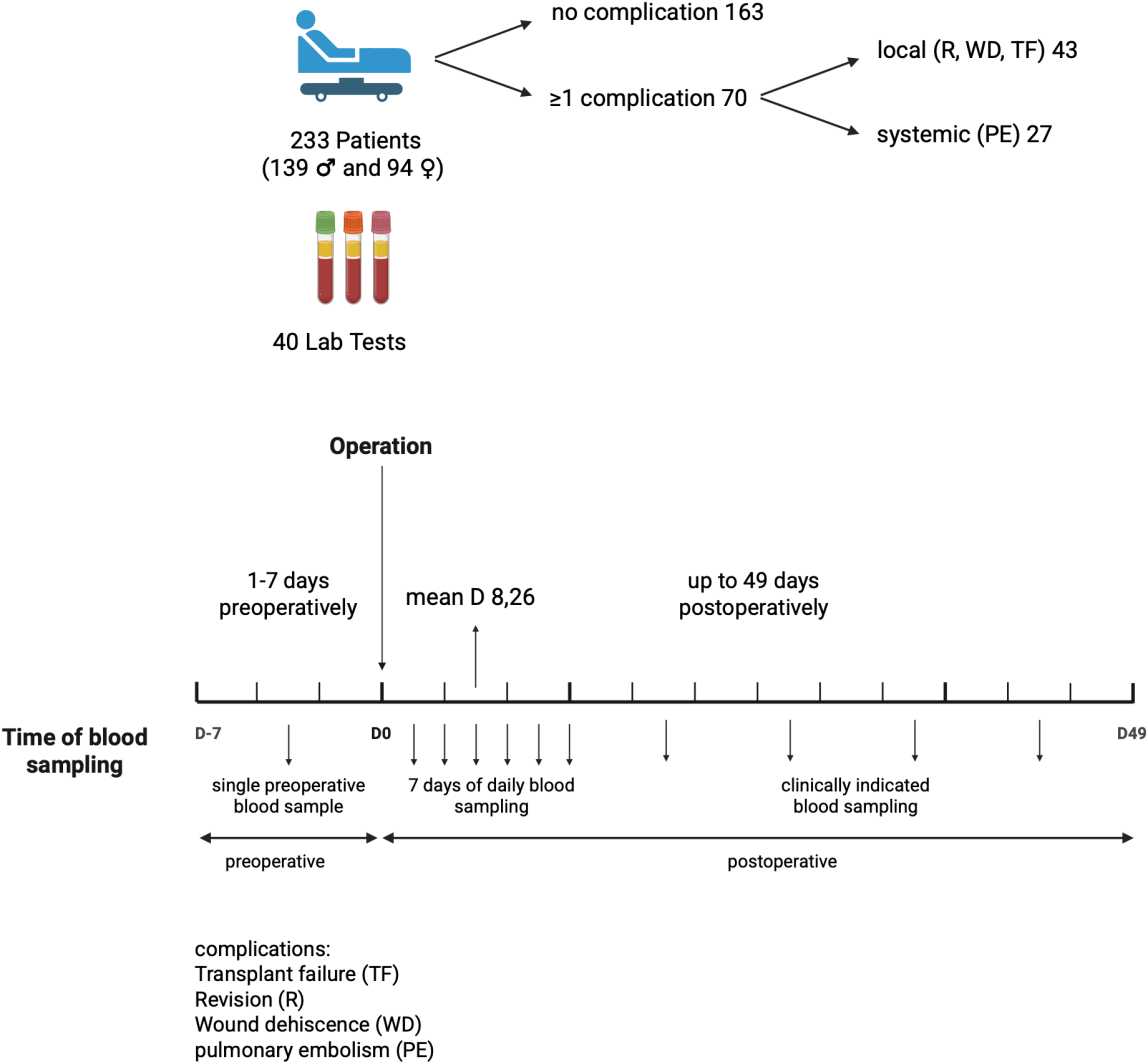
Study design for the laboratory-based evaluation of postoperative complications following head and neck reconstruction surgery in 233 OSCC patients.

This retrospective, single-center cohort study examined 233 treatment-naïve patients with primary OSCC who underwent tumor resection and bone and/or soft tissue reconstruction via local pedicled or free flaps between 2016 and 2023. Four patients had a history of prior radiotherapy unrelated to the tumor site evaluated. Cases of recurrent OSCC were excluded from the analysis. Bone and soft tissue reconstructions were analyzed collectively. Clinical patient characteristics included age at surgery, length of hospital stay, and comorbidities such as diabetes mellitus, nicotine and/or alcohol abuse, peripheral arterial disease (pAVK), depression, and prior radiotherapy. Other characteristics included TNM classification and reconstruction technique, including radial forearm free flap (RFFF), anterolateral thigh flap (ALT flap), fibula free flap (FFF), pectoralis major flap (PMF), osteocutaneous scapula free flap (SFF), nasolabial flap, submental island flap, deep circumflex iliac artery (DCIA) bone flap, and temporal flap, as well as extent of neck dissection. Standard perioperative management included postoperative systemic prophylactic anticoagulation to maintain flap perfusion in patients undergoing free flap reconstruction. Unfractionated heparin (10,000 IU) was administered via an infusion pump as a continuous intravenous infusion over 24 hours, starting six hours after surgery and without an initial bolus. For patients who developed a pulmonary embolism, the prophylactic regimen was escalated to therapeutic, aPTT-adjusted anticoagulation targeting an aPTT value two to three times the patient’s baseline value. Intravenous heparinization continued for one week, after which it was switched to subcutaneous low-molecular-weight heparin for the second postoperative week. Blood sampling and laboratory monitoring were performed as part of routine clinical diagnostic and treatment procedures. Blood samples were obtained once during the week preceding surgery (typically the day before), daily during the first postoperative week, and thereafter as clinically indicated for up to 49 days (**Figure 1**). Patients were informed about these procedures and provided consent as part of their treatment contract. The study was approved by the institutional ethics committee.

### 2.2 Data collection

Clinical and laboratory data were extracted from institutional electronic medical records. The dataset included hematologic parameters (hemoglobin, hematocrit, erythrocytes, leukocytes, platelets, and coagulation tests); renal and hepatic function markers; electrolytes; and inflammatory parameters. All collected parameters are listed in **Table 1**.

**Table 1:** Overview of the laboratory parameters examined and the underlying functional systems.

Function	Parameter
Coagulation	INR
	Fibrinogen
	aPTT
	Antithrombin
	D-dimer
Blood count	Hemoglobin
	Hematocrit
	Erythrocytes
	Platelets
	MCV
	MCH
	MCHC
Electrolytes	Sodium
	Potassium
	Calcium
	Phosphate
Kidney function	Creatinine
	eGFR
	Urea
	Uric acid
Liver function	Bilirubin
	AST (GOT)
	ALT (GPT)
	ALP
	GGT
	LDH
Inflammation	CRP
	PCT
	Leukocytes
Cardiac biomarkers	CK
	CK MB
	Myoglobin
	Troponin I (hs)
	Troponin T (hs)
	NT-proBNP
Proteins	Albumin
	Total protein
Lipids	Triglycerides
Hemolysis	Free hemoglobin
Thyroid gland	TSH

### 2.3 Outcomes

The primary outcomes were defined as postoperative complications, specifically pulmonary embolism (PE), wound dehiscence, the need for surgical revision, and flap loss. For integrative analyses, an overall complication variable (≥1 adverse event per patient) was generated. Subgroup analyses distinguished between the systemic complication PE and local complications, such as wound dehiscence, graft failure, and revision surgery.

### 2.4 Statistics

Descriptive statistics were used to characterize the patient cohort, and complication incidences were reported with 95% confidence intervals. To identify predictive markers, longitudinal laboratory trajectories were summarized by calculating individual linear regression slopes across blood sampling times (preoperatively, daily during the first postoperative week, and thereafter as clinically indicated). These slopes quantified the direction and magnitude of perioperative changes. For some laboratory parameters, such as C-reactive protein (CRP), an increasing slope was considered pathological, whereas for others, such as hemoglobin, a decreasing slope was considered pathological. We performed comparisons between patients with and without complications using Mann-Whitney U test (30). Logistic regression models were applied to evaluate independent predictors of the outcome (31). Both univariable and multivariable analyses were conducted, with model reduction based on the Akaike information criterion (AIC). We reported odds ratios with 95% confidence intervals. Additional analyses addressed the incidence of laboratory values outside the reference range at three time points: preoperatively and during the first and second weeks postoperatively. These analyses used chi-square or Fisher’s exact test, as appropriate (32). Relative changes from baseline to postoperative weeks one and two were calculated and compared using Mann-Whitney U test. As an exploratory complement to classical methods, machine learning techniques were applied (33, 34). Specifically, random forest classification models were constructed to predict the occurrence of complications based on perioperative laboratory dynamics. These models were generated using an ensemble of 500 decision trees with bootstrapped sampling and random selection of predictors at each node. Variable importance was quantified by the mean decrease in the Gini index to determine which laboratory parameters contributed most to predictive performance. Due to the limited number of events and missing values in some parameters, the random forest analyses were limited to proof of concept and were not intended to provide definitive results. The primary outcome parameters of the study were defined as hematocrit, AST, aPTT and INR. These variables were analyzed inferentially using a two-sided significance level of α = 0.05. Additional laboratory parameters were evaluated descriptively; therefore, no adjustment of the significance level for multiple testing was applied. Missing data were neither replaced nor imputed using specific algorithms; they were used as they were, i.e., the values that were available were used, and the number of valid n was provided in each analysis. However, some laboratory values had too many missing data points and were therefore excluded from the analyses. Depending on the number of listwise valid values, the number of missing values reduced the overall patient count in multivariate analyses. Patients with missing values were excluded from the analyses, and the final number of cases is provided. Statistical analyses were performed using R software, version 4.2 (R, 2018; www.R-project.org).

## 3 Results

### 3.1 Cohort characteristics

A total of 233 patients (139 males and 94 females) underwent reconstructive surgery during the study period (Feb. 2016 - Sep 2023). The mean age at surgery was 66.0 ± 12.7 years, and the average hospital stay was 16.9 ± 8.9 days. Of the patients, 47 (20.2%) reported smoking and 26 (11.2%) reported alcohol abuse. Comorbidities were relatively uncommon. Peripheral arterial disease (pAVK) was present in two patients (0.9%), depression in four patients (1.7%), and a history of previous radiotherapy in four patients (1.7%). Regarding tumor staging, T2 tumors were the most prevalent (67 patients, 33.3%), followed by T3 (51 patients, 25.4%) and T1 (48 patients, 23.9%) tumors. Most patients presented with N0 disease (146 patients, 84.4%), while M1 disease was rare (four patients, 1.9%). All clinical patient characteristics are provided in **Table 2**.

**Table 2:** Detailed patient characteristics and clinical outcomes.

Parameter	n (%) or Mean ± SD
Age at surgery [years]	66.0 ± 12.7
Gender Male Female	139 (60%) 94 (40%)
Hospital stay [days]	16.9 ± 8.9
Nicotine abuse No Yes	186 (79.8%) 47 (20.2%)
Alcohol abuse No Yes	207 (88.8%) 26 (11.2%)
Peripheral arterial disease No Yes	231 (99.1%) 2 (0.9%)
Depression No Yes	229 (98.3%) 4 (1.7%)
Previous radiotherapy No Yes	229 (98.3%) 4 (1.7%)
T-stage T0 T1 T2 T3 T4	23 (11.4%) 48 (23.9%) 67 (33.3%) 51 (25.4%) 12 (6.0%)
N-stage N0 N1	146 (84.4%) 27 (15.6%)
M-stage M0 M1	209 (98.1%) 4 (1.9%)
L-stage L0 L1	216 (92.7%) 17 (7.3%)
V-stage V0 V1	232 (99.6%) 1 (0.4%)
Pn-stage Pn0 Pn1	221 (94.8%) 12 (5.2%)
Grading G0 G1 G2 G3	50 (21.5%) 32 (13.7%) 131 (56.2%) 20 (8.6%)
Resection status R0 R1	225 (97.0%) 7 (3.0%)
Distribution of flap types RFFF ALT flap FFF SFF DCIA Local pedicled flaps	88 (37.8%) 35 (15.0%) 44 (18.9%) 16 (6.9) 2 (0.9) 48 (20.6)
Mortality Alive Dead	228 (97.9%) 5 (2.1%)
Total patients	233

### 3.2 Postoperative complications

**Figure 2:**
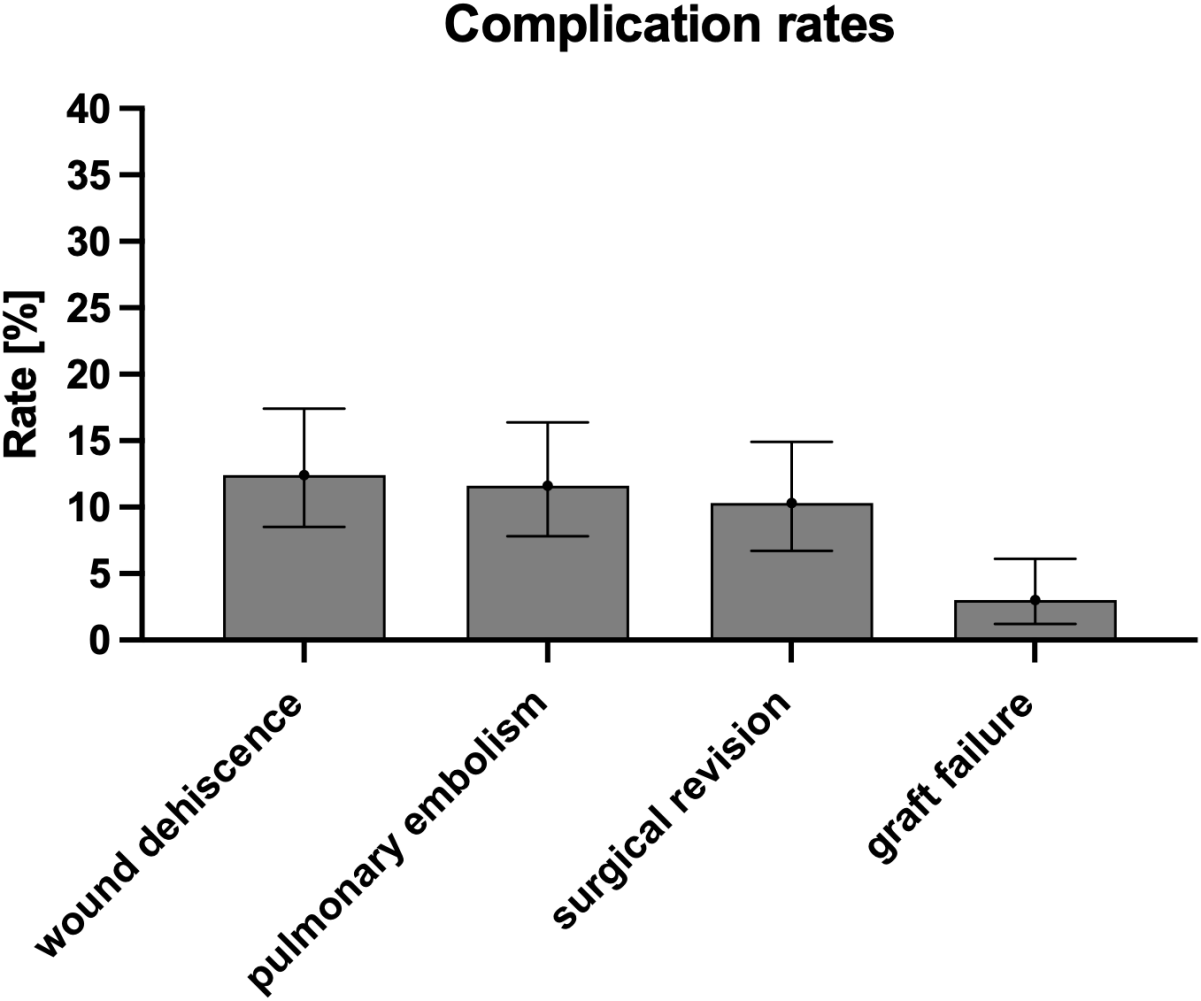
Key findings from statistical analysis of complications in tissue transfer patients (n=233).

The overall complication rate was 30.0% (95% CI: 24.2%-36.4%), affecting 70 out of 233 patients. Specific complications were observed in the following numbers of patients: wound dehiscence (29 patients, 12.4%, 95% CI: 8.5-17.4%), pulmonary embolism (27 patients, 11.6%, 95% CI: 7.8-16.4%), and surgical revision (24 patients, 10.3%, 95% CI: 6.7-14.9%). Graft failure occurred in 7 patients (3.0%, 95% CI: 1.2-6.1%) (**Table 3**; **Figure 2**).

**Table 3:** Overview of postoperative complications.

Complications	n (%) or Mean ± SD
Pulmonary embolism No Yes	206 (88.4%) 27 (11.6%)
Wound dehiscence No Yes	204 (87.6%) 29 (12.4%)
Surgical revision No Yes	209 (89.7%) 24 (10.3%)
Graft failure No Yes	226 (97.0%) 7 (3.0%)

### 3.3 Temporal laboratory trajectories (slopes)

Based on the longitudinal trajectories of laboratory values, a simple linear regression slope was calculated for each patient. This slope served as a quantitative measure of temporal trends in the respective laboratory parameter, indicating stability (slope = 0), an increase (slope > 0), or a decrease (slope < 0) over time. Slope profiles were compared between patients with and without complications to assess whether temporal changes in laboratory values could predict the occurrence of complications. Patients with complications showed less negative day-to-day slopes compared to patients without complications. Significant differences were observed in hemoglobin, hematocrit, erythrocytes, and total bilirubin levels (see **Table 4**).

**Table 4:** Markers with significant slope differences (any complication vs none).

Parameter	No complications (n=163)	Complications (n=70)	P-value
Hemoglobin slope	-0.18 ± 0.44	-0.10 ± 0.15	0001
Hematocrit slope	-0.54 ± 0.69	-0.34 ± 0.53	0.011
Erythrocyte slope	-0.10 ± 0.43	-0.05 ± 0.16	0.007
Total bilirubin slope	-17.15 ± 168.2	-0.67 ± 4.23	0.01

#### 3.3.1 Predictive modeling

In univariable models, hematocrit slope (OR: 1.74; 95% CI: 1.03–2.92; p = 0.04) and AST slope (OR: 1.12; 95% CI: 1.01–1.23; p = 0.02) were significant predictors. In a reduced multivariable model, the hematocrit slope remained a significant predictor (OR: 4.02; 95% CI: 1.18–13.64; p = 0.03). Overall performance: McFadden pseudo-R² = 0.080, accuracy = 0.71 (95% CI = 0.64–0.77), TPR = 0.16, TNR = 0.97, PPV = 0.73, NPV = 0.71 (**Table 5**).

**Table 5:** Logistic regression predictors (per-day slope).

Parameter	Odds ratio	95% CI	P-value	Model
Hematocrit slope	1.74	[1.03-2.92]	0.04	Univariable
AST slope	1.12	[1.01-1.23]	0.02	Univariable
Hematocrit slope	4.02	[1.18 – 13.64]	0.03	Reduced multivariable

**Figure 3:**
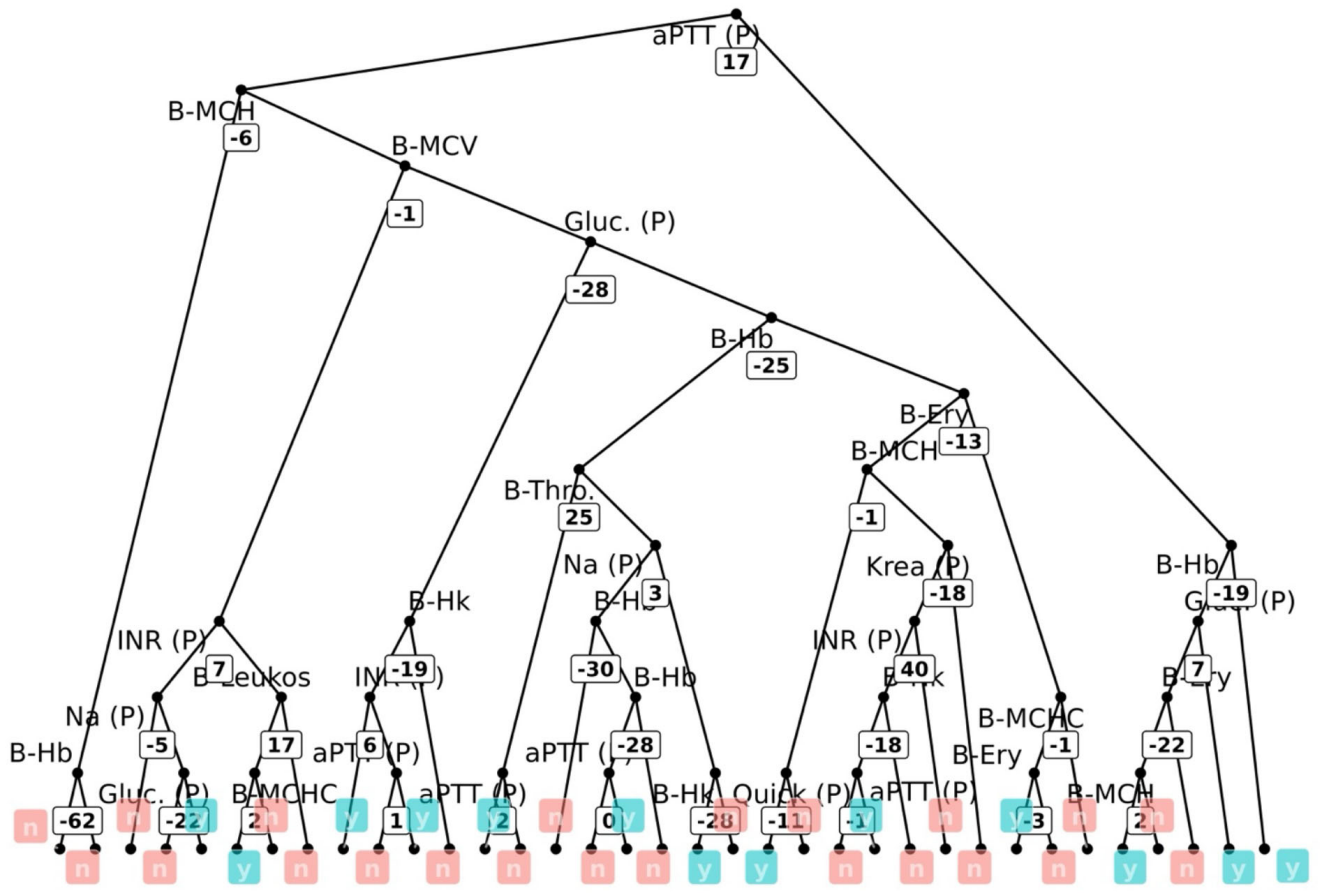
Random forest plot of the 2-week values for predicting complications (in turquoise: complication y = yes, in red n = no, numbers in boxes: threshold value of the respective % change value from baseline).

To capture nonlinear interactions among multiple laboratory changes, a random forest was trained using a 1:1 train-test split on percent change from baseline to postoperative week 2. The variable importance ranking (by mean Gini index) identified aPTT and INR as the most predictive features, followed by MCHC and sodium (**Table 6**). **Figure 3** provides an illustrative decision tree with threshold values.

**Table 6:** Top random-forest features ranked by mean Gini importance.

Feature	Rank
aPTT	1
INR	2
MCHC	3
Sodium (Na)	4

#### 3.3.2 Deviations from reference ranges (incidence over time)

During the preoperative and weeks 1 and 2, complications were associated with higher incidences of abnormal aPTT (weeks 1–2), platelets (week 2), leukocytes (week 2), potassium (week 2), creatinine (week 1), reduced eGFR (week 1), elevated urea (preoperative and week 1), low total protein (preoperative and week 1), and elevated ALT (preoperative) (**Table 7**).

**Table 7:** Selected abnormality incidences associated with any complication.

Parameter	No complications (%)	Complications (%)	P-value
aPTT (week 1)	38	70	0.001
aPTT (week 2)	20	42	0.002
Platelets (week 2)	67	86	0.006
Leukocytes (week 2)	38	57	0.015
Potassium (week 2)	49	74	0.001
Creatinine (week 1)	49	69	0.007
eGFR (week 1)	14	35	0.015
Urea (preop)	18	32	0.046
Urea (week 1)	25	41	0.026
Total protein (preop)	11	22	0.049
Total protein (week 1)	94	100	0.042
ALT (preop)	6	17	0.010

#### 3.3.3 Pre-post changes from baseline

Percent changes from baseline to the first postoperative week revealed differences in platelet count, AST, MCV (borderline), and TSH (**Table 8**).

**Table 8:** Selected percent changes (baseline → week 1).

Parameter	Windows	P-value
Platelets	baseline → week 1	0.011
AST	baseline → week 1	0.034
MCV	baseline → week 1	0.050
TSH	baseline → week 1	0.011

#### 3.3.4 Systemic vs local complications (exploratory stratified analyses)

Of the 70 patients who experienced complications, 27 (38.6%) had the systemic complication PE. Meanwhile, 43 (61.4%) had local complications. Several laboratory trajectories and incidences of abnormality differed by complication type (**Tables 9**, **10**).

**Table 9:** Slope differences (systematic vs local complications).

Parameter (per-day slope)	Comparison	P-value
INR	Systemic vs local	0.042
aPTT	Systemic vs local	0.004
Potassium	Systemic vs local	0.045
Urea	Systemic vs local	0.038
Total protein	Systemic vs local	0.002

**Table 10:** Selected abnormality incidences and percent changes (systemic vs local complications).

Parameter (timepoint)	Comparison	P-value
aPTT (preop, week 1, week 2)	Systemic higher	0.023/0.024/0.009
Sodium (week 2)	Differential	0.008
Phosphate (preop, week 1)	Differential	0.025/0.008
TSH (preop)	Differential	0.040
aPTT (baseline→week 1)	Systemic vs local	0.006
Hemoglobin (baseline→week 1)	Systemic vs local	0.030
Hematocrit (baseline→week 1)	Systemic vs local	0.031
TSH (baseline→week 1)	Systemic vs local	0.024
INR (baseline→week 2)	Systemic vs local	0.004
Fibrinogen (baseline→week 2)	Systemic vs local	0.003
aPTT (baseline→week 2)	Systemic vs local	0.002

## 4 Discussion

In this single-center retrospective cohort study of 233 patients undergoing head and neck reconstruction after OSCC resection, perioperative laboratory dynamics emerged as valuable predictors of postoperative complications. While the 30% overall complication rate in our study is lower than the 36.1% - 54% range reported by others (15, 35–37), it still highlights the significant morbidity associated with complex reconstruction in this anatomically challenging region.

A distinctive feature of our work is our emphasis on longitudinal trends rather than static values. Patients who developed complications exhibited less pronounced declines (i.e., less negative slopes) in hemoglobin, hematocrit, erythrocytes, and total bilirubin. Slope-based hematocrit retained predictive value in regression analyses. Analyses of change from baseline were particularly informative, suggesting that temporal trajectories carry greater prognostic content than single time points. This motivates continuous, early postoperative monitoring for an appropriate amount of time (e.g., two weeks).

The observation of a flatter negative hemoglobin slope in patients with complications contrasts with previous studies reporting that low preoperative hemoglobin levels are associated with flap failure, thrombosis, transfusion requirements, and adverse outcomes (38–42). In particular, threshold values of approximately 10–12 g/dl have been linked to an increased risk of flap loss, thrombotic events, and the need for transfusion (38–41), and a postoperative hemoglobin decrease of more than 3.8 g/dl has been shown to further worsen prognosis (42). However, the comparatively smaller decrease in hemoglobin in patients with complications in our cohort can mainly be explained by a higher transfusion frequency in this group and indicates the need for adequate oxygen supply to the tissue, which should be considered in perioperative management.

Beyond erythrocyte indices, identifying total bilirubin as a candidate marker meaningfully broadens conventional assessment. Elevated total bilirubin, particularly in conjunction with AST, likely reflects an integrated response to perioperative tissue injury, hypoxia, hemolysis, and limited hepatic reserve, thereby capturing subclinical hepatic dysfunction that is not apparent from erythrocyte indices alone. Elevated liver AST has been associated with higher overall complications and surgical-site infections (43), and advanced liver disease correlates with increased postoperative morbidity and prolonged length of stay (44). Moreover, postoperative cell death from tissue hypoxia or surgical trauma may cause concurrent elevations in total bilirubin and AST, as cell lysis releases intracellular AST and hemoglobin, which is subsequently degraded to bilirubin (45). Together, these observations support incorporating dynamic perioperative bilirubin and AST trajectories into risk stratification algorithms to enable earlier identification of patients at increased risk for complications and to guide more individualized perioperative management.

Coagulation and electrolyte signals also emerged as clinically relevant. Dynamic abnormalities in INR and aPTT, as well as perioperative electrolyte disturbances - especially hyponatremia - were associated with adverse outcomes. This is consistent with literature linking electrolyte disorders to higher complication and infection rates (43). Notably, most studies have not found INR/aPTT to predict bleeding outside of hepatic surgery (46). In our context, therefore, these coagulation markers should be interpreted primarily as proxies of systemic stress and thromboinflammation rather than direct predictors of bleeding. Dynamic abnormalities in INR/aPTT and sodium likely reflect systemic inflammation, neurohormonal stress, and hepatic or endothelial dysfunction rather than isolated defects in hemostasis or water balance. Accordingly, future perioperative pathways could integrate coagulation and electrolyte trajectories into risk stratification algorithms, using persistent deviations as triggers for closer monitoring, targeted correction, and early investigation of sepsis, organ dysfunction, or thromboinflammatory complications.

Importantly, our cohort provides a rationale for the different coagulation profiles observed in systemic versus local complications. All flap patients received preventive postoperative heparinization to secure flap perfusion. This was therapeutically escalated in case of pulmonary embolism (PE), explaining the higher aPTT levels in this subgroup. Thus, increased aPTT serves as a management signal rather than an intrinsic pre-event risk marker. In contrast, INR was altered preoperatively in patients who later developed PE, diverging further thereafter. These patterns are not explained by the heparinization and support a biological interpretation involving tumor- and surgery-related thromboinflammation (47), hepatic congestion/hypoxia, or subclinical hepatic dysfunction (48) as well as a cancer-associated procoagulant state (49). Taken together, coagulation tests in this setting are best viewed as complementary. aPTT largely reflects treatment intensity (50), whereas INR, partly altered before any therapeutic escalation, may index baseline vulnerability and early systemic stress (51).

Methodologically, coupling conventional statistics with an exploratory random forest screen reflects the broader shift toward machine learning support in perioperative medicine (52). Contemporary work indicates that such algorithms can outperform traditional scores in predicting complications (53), and in our dataset, the machine learning signals converged with classical analyses to help prioritize candidate features. Clinically, these findings complement and extend existing risk stratification tools. While the HALP score (hemoglobin, albumin, lymphocytes, and platelets) has demonstrated strong discrimination for complications in head and neck reconstruction (area under the curve [AUC] ≈ 0.85) (54), our results add hepatobiliary dimensions (bilirubin and aspartate aminotransferase [AST]) and trend-based coagulation and electrolyte features that capture the physiological response to surgical stress more directly than static indices. The practical implication is a structured perioperative surveillance strategy. Integrating automated trend analytics into the electronic health record could enable real-time early warning systems, particularly during the first postoperative week, when the richest prognostic signal was observed in our cohort.

However, these strengths must be weighed against important limitations. The retrospective, single-center design limits generalizability and invites residual confounding. The relatively low frequency of individual complications necessitated the use of a composite endpoint, which restricts phenotype-specific inference and could dilute the signal. Class imbalance and missing data across laboratory panels reduced sensitivity and precluded stable multivariable or ensemble models. Additionally, measurement timing was not fully standardized, and slope estimates assume approximate linearity between irregular time points. These estimates may be influenced by fluid shifts, transfusions, and sampling schedules (55). Therapeutic confounding is significant: despite universal heparinization, aPTT elevations in the PE group are primarily treatment-driven, whereas INR trajectories - already divergent preoperatively - are not heparin-mediated and more accurately reflect systemic pathophysiology. Finally, we lacked granular, time-varying covariates for anticoagulation intensity (e.g., UFH targets and anti-Xa levels), vitamin K status, antibiotics, nutrition, and high-resolution operative variables. Event timing was not uniform enough to model pre-event slopes for every case. Additionally, tumor burden and systemic inflammation may confound both PE risk and laboratory behavior.

## 5 Conclusions

This study highlights the potential of laboratory-based risk stratification, especially when leveraging temporal patterns, while demonstrating that multiple interacting influences shape these signals. Therefore, the data should be regarded as hypothesis-generating. They identify candidate domains (e.g., erythrocyte indices, hepatobiliary markers, coagulation, and electrolytes) and operational contours (e.g., early, trend-aware monitoring). However, they also emphasize the need for prospective, multicenter validation with event-centered sampling, standardized laboratory schedules, and time-varying adjustment for therapy and supportive care. Future trials should pre-specify actionable thresholds, evaluate calibration and clinical utility (e.g., decision curve analysis), and test generalization across centers and techniques so that laboratory trajectories can be translated into reliable early warnings and targeted interventions that improve outcomes after complex head and neck reconstruction.

## Data Availability

The datasets presented in this study can be found in online repositories. The names of the repository/repositories and accession number(s) can be found below: https://doi.org/10.25625/0VOVNJ.
